# Experimental Evidence for the Missing and Theoretically Predicted [5,5] C_150_-D_5h_ Fullertube and Isolation of a Second C_150_ Isomer

**DOI:** 10.1021/acsomega.6c06857

**Published:** 2026-10-25

**Authors:** Xiaoyang Liu, Kevin Cover, Rong Huang, Ryan M. Koenig, Hannah M. Franklin, Xu Feng, Steven Stevenson, Harry C. Dorn

**Affiliations:** † Department of Chemistry, 1757 Virginia Polytechnic and State University, Blacksburg, Virginia 24060, United States; ‡ Department of Chemistry and Biochemistry, 14688 Purdue University Fort Wayne, Fort Wayne, Indiana 46805, United States; § FIRST Molecules Research Center, Purdue University Fort Wayne, Fort Wayne, Indiana 46805, United States

## Abstract

Novel allotropes of elemental carbon (e.g., fullerenes, carbon nanotubes, and graphene) are exciting. In 2019, nanotubular fullerenes (fullertubes) were experimentally discovered as a family of segmental, tubular molecules. We report the longest fullertube isolated to date, from 130 atoms in 2023 to now 150 carbon atoms. We have purified two structural isomers of C_150_. The late-eluting C_150_ species (Isomer II) represents the largest soluble fullertube/fullerene pristine molecule successfully isolated. From a total pool of 335,568 isolated-pentagon rule (IPR) candidate structures, surprisingly only three fullertube possibilities emerge. Isomeric purification of C_150_ was achieved via chemical selectivity (Stage 1) and HPLC (Stage 2). Isolated C_150_ fullertubes were characterized via mass spectrometry, XPS, Raman, and anisotropic polarizabilities and compared with DFT computations. Consistent with an established paradigm in the literature for fullertube structural assignments, we correlated chromatographic data (ln capacity factor, k′) in a plot of experimental HPLC data versus DFT-calculated anisotropic polarizabilities. Results indicate Isomer I is indistinguishable between [9,0] C_150_-D_3h_ or [9,0] C_150_-D_3_. However, for Isomer II, multiple measures of experimental evidence (combined with DFT calculations) both support the decades-missing [5,5] C_150_-D_5h_ fullertube structure long predicted by the literature. Plainly stated, the D_5h_ assignment of a C_150_ fullertube structure is a strong prediction pending direct imaging confirmation with atomic resolution TEM.

## Graphical abstract

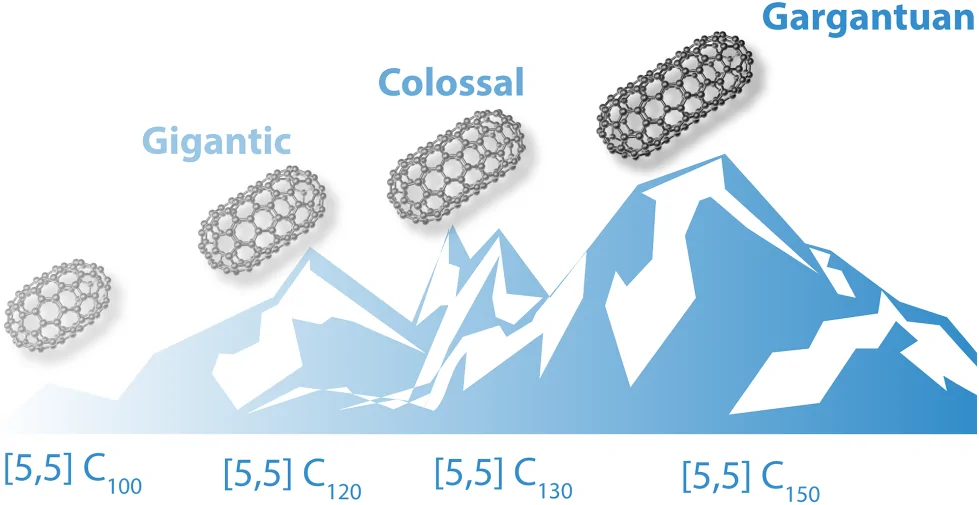

## Introduction

Since the discovery of nanotubes nearly 4 decades ago, single-walled carbon nanotubes (SWCNTs) have acquired considerable scrutiny due to the immense number of potential applications of these 1D materials.^1,2^ However, applications for these new 1D SWCNT materials are beset by the absence of highly purified SWCNTs of a reproducible length, diameter and chirality.^3–8^ For the case of *fullertubes*, which are, in reality, fullerene-based end-capped SWCNTs.

The isolation of new forms of a soluble molecular carbon *family* (fullertubes) were experimentally first reported in 2019.^9^ Fullertubes are pristine molecules formed from linking two structural motifs, rolled graphene of single-walled carbon nano *tubes* (SWCNTs) and hemisphere end-caps of *fuller* enes, Figure 1.

**Figure 1:**
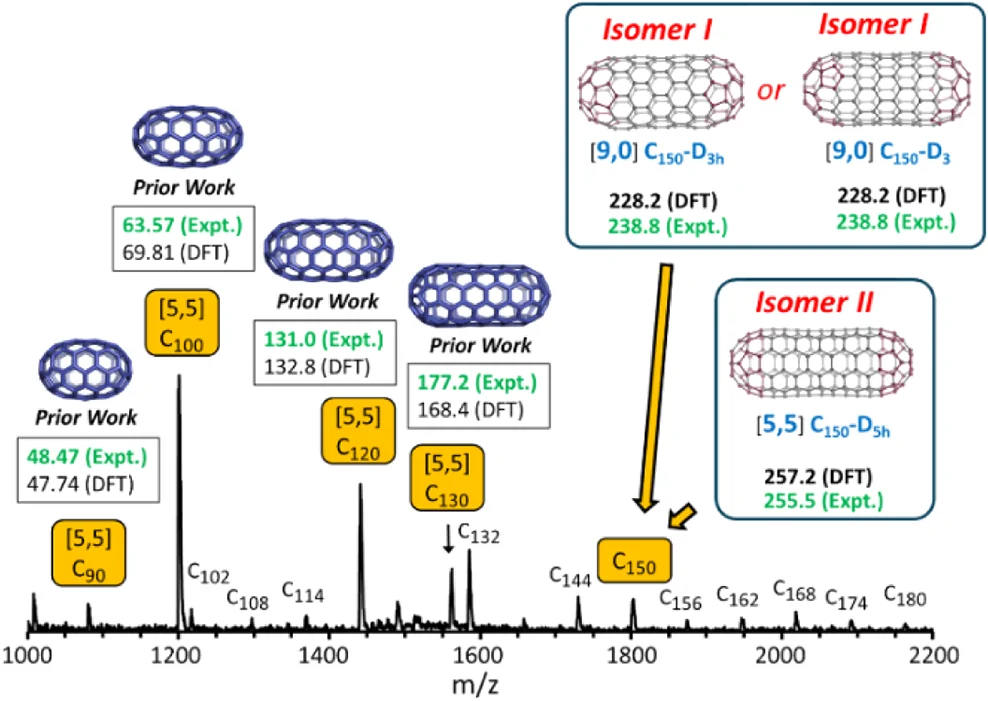
Literature precedence for prior fullertube structural assignments using the paradigm of correlating anisotropic polarizability DFT values versus chromatographic capacity factor (k′) experimental values.

Chromatographic retention times (t_r_) of fullertubes are consistent with strong van der Waals electron interactions between fullertubes and the chromatographic stationary phase/fullertube π–π interactions.^10–12^ We can structurally predict and distinguish between [9,0] versus [5,5] C_150_ candidate fullertube isomers using a now established literature method of plotting chromatographic retention data versus anisotropic DFT polarizability obtained from calculations.^10–12^

## Computational Methods

All DFT calculations were performed using Gaussian 09 at the B3LYP/6-31G­(d) level. Candidate isomers underwent full, unconstrained geometry optimization with default convergence criteria. Dispersion corrections were omitted, as this theory level is well-benchmarked for such isolated, rigid cages. Subsequent single-point calculations yielded the HOMO–LUMO gaps. See SI for complete details.

## Results and Discussion

The paradigm of correlating polarizability versus chromatographic retention was first employed to understand subtle chromatographic retention differences in a family of 16 members of the A_3_N@C_80_-I_h_ and the A_3_N@C_80_-D_5h_ trimetallic nitride endohedral metallofullerenes.^13^ More recently, Bourret reported on this new structural identification approach based on chromatographic retention data versus polarizability (and especially anisotropic polarizability).^14^ The Bourret model clarified the influence of tubular versus spheroidal and ellipsoidal shapes when predicting retention behavior for a large array of fullerenes, fullertubes, and polycyclic aromatic hydrocarbons (PAHs).^14^

Fowler and Manolopoulos have shown an alternating 10 carbon symmetry sequence that is exhibited for tubular fullerenes, D_5h_ → D_5d_ → D_5h_ → D_5d_ → D_5h_ → D_5d_ → D_5h_.^15^ That is to say, this new structural elucidation approach via anisotropic polarizability has accurately retrospectively predicted all four reported fullertube structures (e.g., X-ray, HR-TEM (High Resolution-Transmission Electron Microscopy), STM (Scanning Tunneling Microscopy) for [5,5] C_90_-D_5h_, [5,5] C_100_-D_5d_, [5,5] C_120_-D_5d_ and [5,5] C_130_-D_5h_ members of the [5,5] fullertube family). This is illustrated by the remarkable data predictive agreement between DFT polarizabilities and chromatographic retention as shown in Figure 1.^10–12^

Since 4 members of this alternating symmetry [5,5] sequence have already been reported, it is easy to predict that C_140_, and C_150_ should have D_5d_ and D_5h_ symmetry isomers, respectively.^15^ Hence, this work represents the fifth usage of this established, structural assignment paradigm, from C_90_ and C_100_ (2020, 2021),^16,17^ C_120_ (2022),^11^ C_130_ (2023),^10^ and, herein, C_150_ (2026).

Herein, we present evidence for successfully isolating *two* of the longest C_150_ fullertubes from an array of 335,568 stable isolated pentagon rule (IPR) candidate isomers.^15^ For the [5,5] C_150_ isomer, there are a total of 130 π tubular belt carbons. These π carbons represent the SWNT moiety of the fullertube molecule, (see Figure 1).

A mass spectral comparison of the initial carbon soot extract (Figure 2a) versus the carbon species surviving a chemical attack with 3-aminopropanol, NH_2_PrOH (Figure 2b) indicates a successful and facile approach for obtaining samples of highly enriched fullertubes. See SI for reaction details. Note the significant removal of C_76_–C_200_ higher fullerenes (Figure 2a) and large reduction of spheroidal C_70_ (Figure 2b). After HPLC (Figure 2c, d), a high purity of two C_150_ isomers is confirmed (Figure 2e-h). The observed [5,5] fullertube family members (i.e., C_60+10n_) have survived the harsh aminopropanol chemical separation protocol as a separate carbon class due to their lower chemical reactivity.^16^

**Figure 2:**
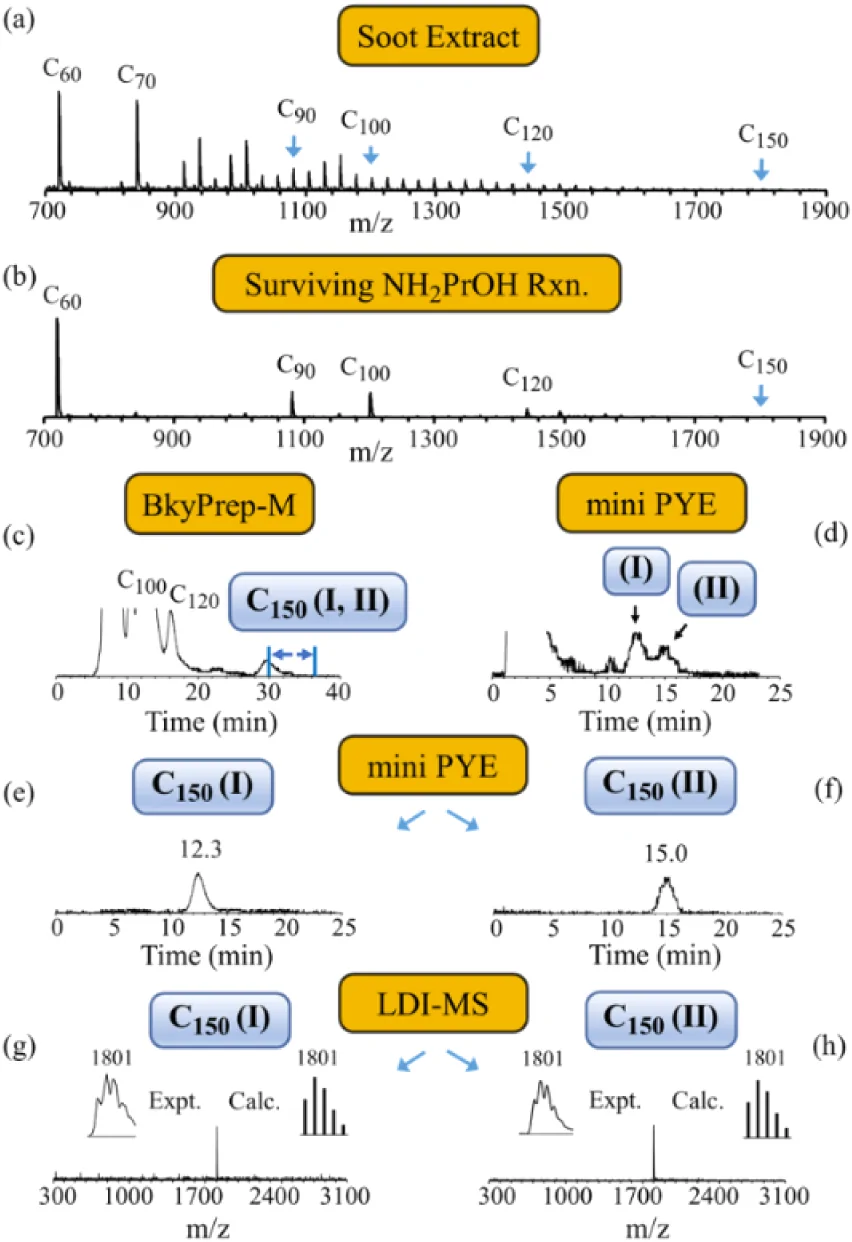
LDI mass spectra of (a) carbon soot extract, (b) species remaining in solution after NH_2_PrOH addition, (c) Chromatogram of (b) on a semiprep BuckyPrep-M, 1.0 mL/min *o*-xylene. (d) Chromatogram after injection of the C_150_ region shown in (c) but injected into a miniPYE analytical column (2.0 mm × 150 mm). See SI. Final chromatograms and LDI mass spectra for purified isomer I, either [9,0] C_150_-D_3_ or [9,0] C_150_-D_3h_, (structure undetermined between these two D_3_ candidates) shown in (e, g), and (f, h) isomer II, [5,5] C_150_-D_5h_, 0.5 mL/min *o*-xylene.

To unambiguously demonstrate (1) the *elemental* purity of the two purified C_150_ isomers, 12.3 min. (I) and 15.0 min. (II) and a (2) successful removal of organic contaminants prior to subsequent characterization experiments, we obtained the XPS spectra shown in Figure 3. In stark contrast with XPS results for most SWCNTs reported to date,^18^ the C_1s_ XPS spectral binding energy peak for isolated [5,5] C_150_-D_5h_ is highly symmetric and narrow at 284.8 eV (fwhm, Full Width Half Height at 1.0 eV) (Figure 3) and indicates a high elemental carbon purity. This assignment of high purity elemental carbon is consistent with the absence of significant C–O bond formation and only very minor Au surface oxygen surface contamination of the O_1s_ peak that would have been centered at ∼530 eV (Figure 3).

**Figure 3:**
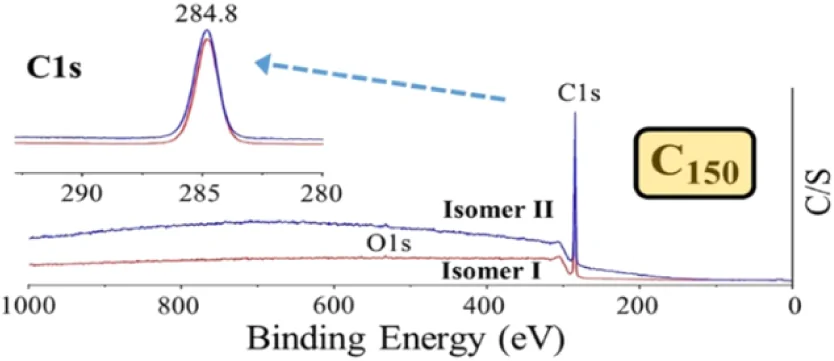
XPS spectra and C purity of purified C_150_ isomers.

The next step toward structural characterization is based on downsizing the large pool of isolated pentagon rule (IPR) candidate isomers based on DFT property and structure calculations. Utilizing the Fowler and Manolopoulos spiral algorithm, the number of potentially stable isolated pentagon rule (IPR) candidate isomers is 335,568 C_150_ with corresponding symmetries detailed in the SI.^15^

Their spiral algorithm predicts 17 candidate IPR isomers of C_150_ possible structures with 3, 5, and 6-fold symmetry, but only 3 C_150_ candidate isomers have tubular symmetry (Figure 4). The rationale for limiting DFT calculations to only 3, 5, and 6-fold isomers has been previously described.^11^ It should be noted that 2-fold isomers should also be included in this criteria, but there are no D_2_ isomers of C_150_ that are fullertube in structure (SI). Not observing any D_2_ fullertube candidate isomers from these 335,568 possible IPR satisfying isomers is nontrivial. This structural enumeration result for D_2_ candidates was verified computationally and using the Fowler-Manolopoulos spiral algorithm.^15^ Further details (e.g., DFT calculations, HOMO–LUMO gaps, xyz coordinates, anisotropic and average polarizabilities) for all 17 of these isomers are provided in the SI.

**Figure 4:**
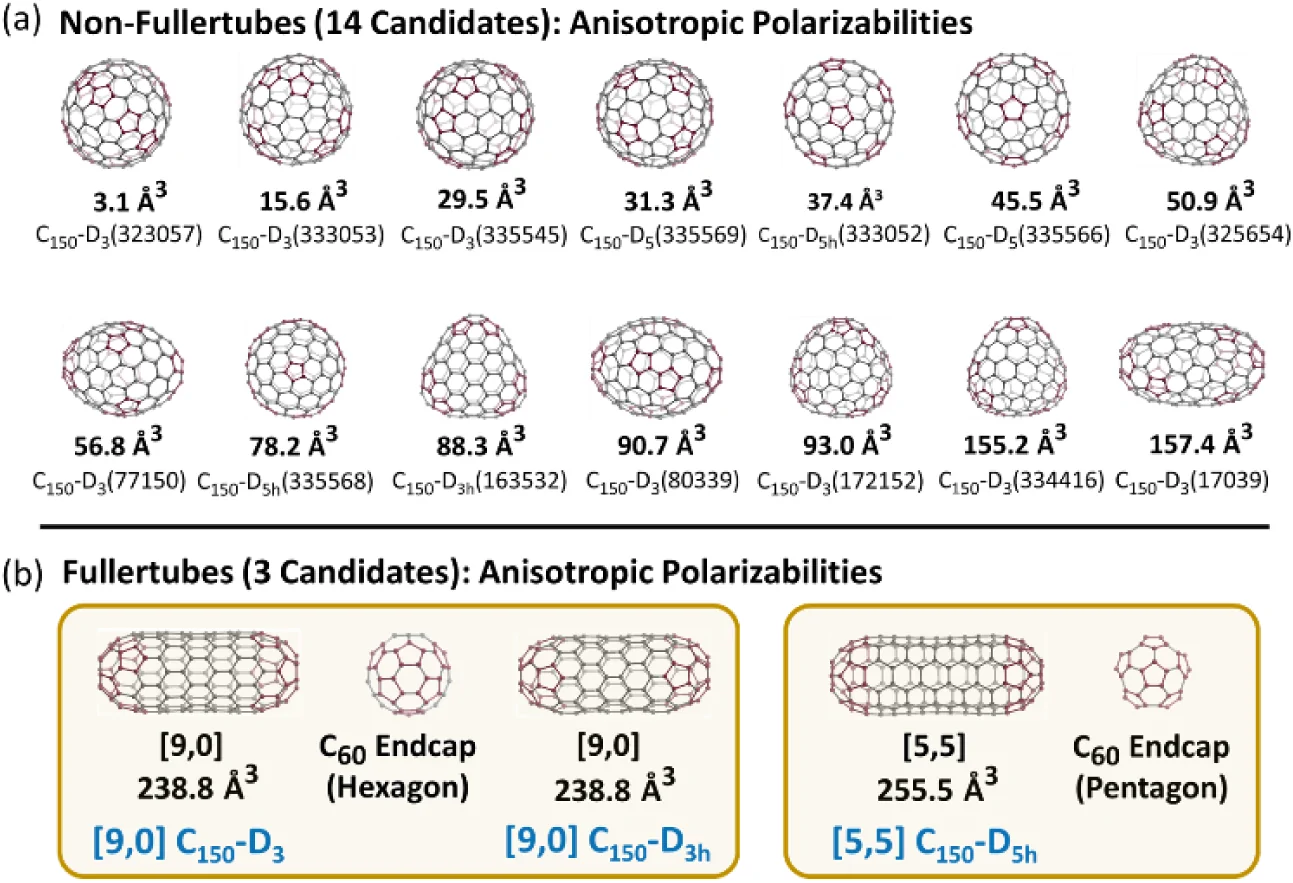
Down selection from 335,568 possible isomer candidates into only 3, 5, and 6-fold symmetry structures: (a) 14 spheroid/ovoid and (b) 3 fullertube candidates.

A key strategy in the structural assignment paradigm of down selecting candidate C_150_ isomers is the chromatographic elution time versus DFT calculated anisotropic polarizability. Based on reported findings to date, [5,5] C_90_-D_5h_,^16,17,19^ [5,5] C_100_-D_5d_,^16,17^ [5,5] C_120_-D_5d_ ^11^ and [5,5], C_130_-D_5h_,^10^ a [5,5] C_150_-D_5h_ isomer is anticipated *a priori* in the current study. For the chromatographic peak at 15 min, we logically propose a structural assignment of [5,5] C_150_-D_5h_ to isomer II using the established method of plotting chromatographic retention data versus anisotropic DFT polarizability (Figure 5).

**Figure 5:**
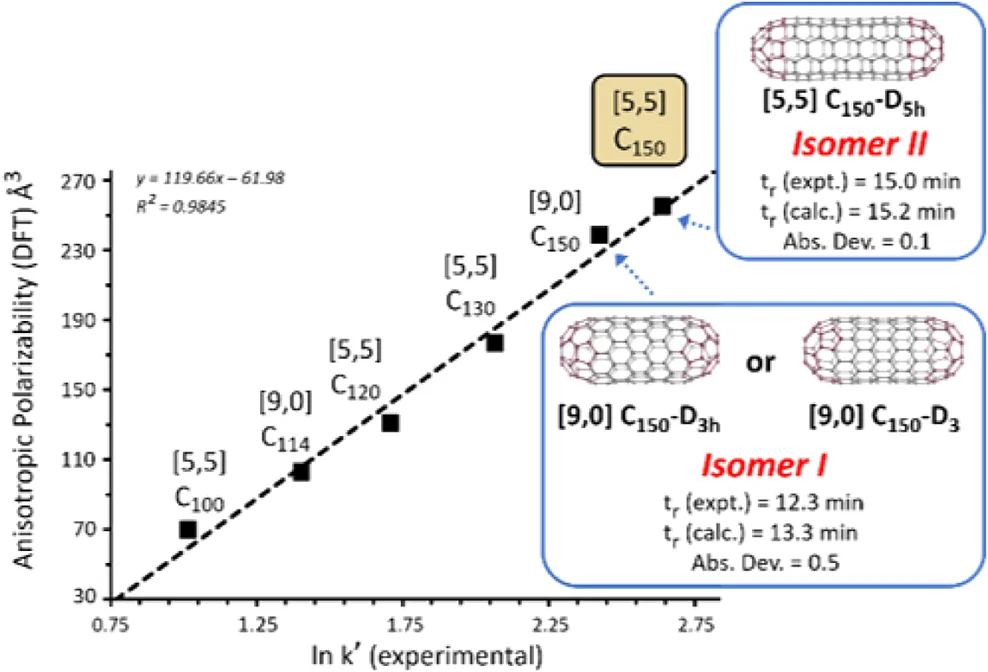
Graphical correlation of DFT anisotropic polarizability (Å^3^) versus the natural log (ln k′) of experimentally obtained capacity factors for structural predictions of candidate C_150_ isomers. Note: k′ = (t_r_ – t_o_)/t_0_.

Among the 17 isomers with 3, 5, and 6-fold symmetry (Figure 4 and SI), only the 15 min [5,5] C_150_-D_5h_ candidate isomer successfully fits the data in Figure 5 with a matching (and largest) DFT calculated anisotropic polarizability (255.5 Å^3^). Note the almost identical retention times upon comparing 15.2 (theoretical) versus 15.0 min (experimental). Possessing the largest anisotropic polarization value results in the last eluting HPLC peak at 15 min (Figure 2d). The higher the anisotropic polarization, the larger the ratio of fullertube length to diameter. This is helpful in predicting the longest retention time of eluting peaks since the elution order increases in proportion to anisotropic polarizability. This ability to correctly assign structural isomers based on anisotropic polarizability versus retention time is consistent with accurate structural assignments to fellow [5,5] C_90_, C_100_, C_120_, and C_130_ fullertube family members reported to date.^12^

Based on the graph in Figure 5, it is clearly not possible to distinguish between the two [9,0] candidate structures because of their identical values of anisiotropic polarizability. Fortuitously for the C_150_ Isomer II species, there are *no* alternative [5,5] candidate structures. The anisotropic polarizability graph (Figure 5) clearly shows isomer II as the sole possibility in its structural prediction as [5,5] C_150_-D_5h_ since no competing structures exist for this retention time. While the inclusion of empirical dispersion corrections or diffuse functions would shift the absolute polarizability values, benchmarking on rigid fullerene cages demonstrates that relative polarizabilities are highly conserved across methods. Because this assignment relies on a linear calibration trendline, systematic scaling effects from the B3LYP/6–31G­(d) method are internally normalized, ensuring the assignment of Isomer II is not an artifact of the chosen level of theory.

Note the [5,5] C_150_-D_5h_ (Figures 1, 6b) is now the longest fullertube (1.76 nm) and smallest diameter (0.668 nm) reported to date. The Bourret study clearly pointed out that fullerenes and fullertubes possessing long tubular lengths and not having spherical or elliptical shape characteristics exhibit large DFT anisotropic polarizabilities and long chromatographic retention times.^14^ Beyond the nondog bone shape of the tubular region in [5,5] C_120_-D_5d_ (Figure 6a), there is a considerable variation in DFT predicted diameters along the tubular length of the longer [5,5] C_150_ fullertubes (Figure 6b, c). This “dog-bone” type fullertubular structure was predicted for [5,5] SWCNTs by Cioslowski.^20^

**Figure 6:**
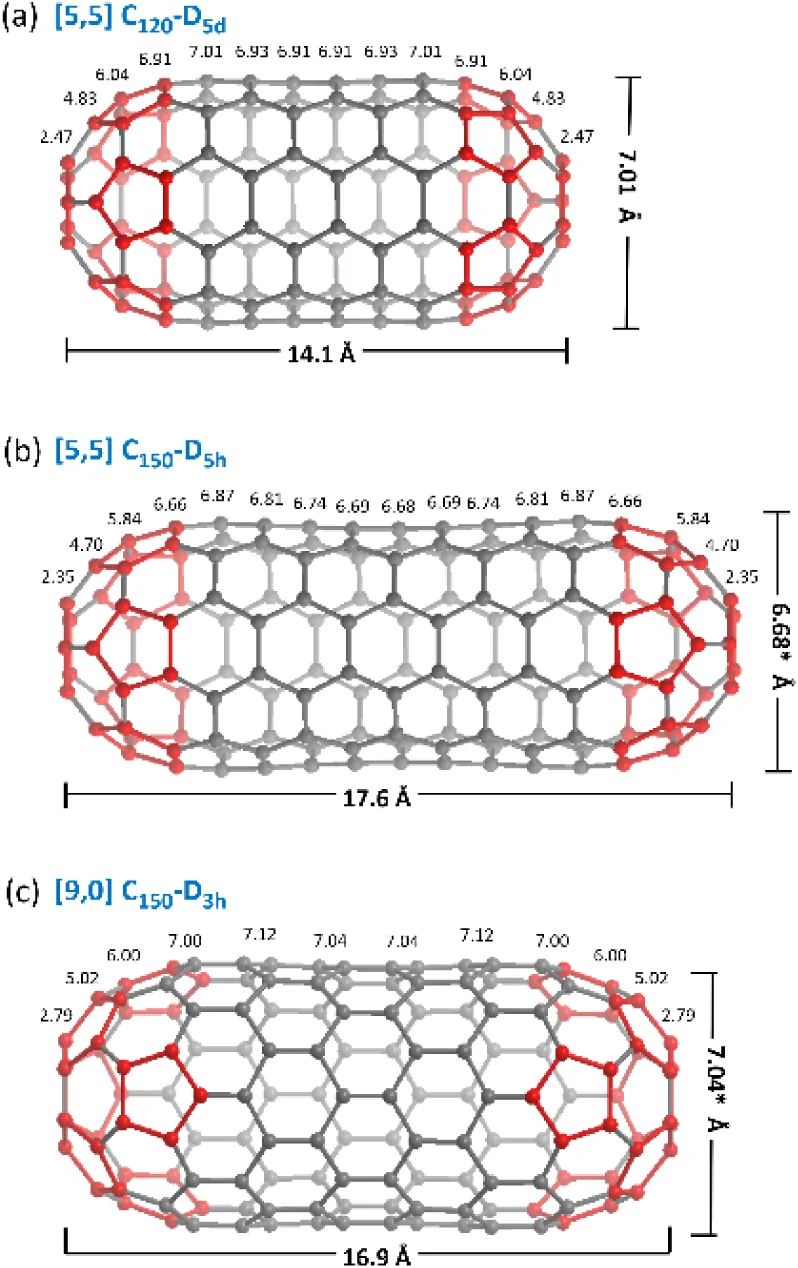
Diameter versus length comparison of (a) [5,5] C_120_-D_5d_, (b) [5,5] C_150_-D_5h_ and (c) [9,0] C_150_-D_3h._ The asterisks in (b) and (c) represent the radius at the middle of the tubular belt.

For the chromatographic peak at 12.3 min (isomer I), the same DFT anisotropic polarizability value (238.8 Å^3^) is calculated for both possible isomers [9,0] C_150_-D_3h_ (Figure 6c) and [9,0] C_150_-D_3_. These two structural isomers possess the *next* highest calculated anisotropic polarizability values in the 3, 5, and 6 symmetry selection, and they correlate with the chromatographic retention value of 12.3 min, Figure 2. Note the end-caps for the two [9,0] isomers originate from a carbon hexagon ring versus a pentagon. Interconversion of the [9,0] C_150_-D_3h_ (Figure 6c) and [9,0] C_150_-D_3_ candidates occurs by slicing the molecule in half and rotating the bottom half 30° about the hexagon end-cap orthogonal to the *C*_*3*_ axis and reattaching the bottom moiety. Furthermore, the diameter at the center of isomer [5,5] C_150_-D_5h_ (Figure 6b) is significantly smaller than both the [9,0] C_150_-D_3h_ and [5,5] C_120_-D_5d_, Figure 6a-c. For the case of [5,5] C_150_-D_5h_ (Figure 6c) it is suggestive of a subtle progression to a more elliptical shape and a smaller calculated anisotropic polarizability value than the corresponding [5,5] C_150_ -D_5h_ isomer, Figure 6b.

In order to distinguish between the [9,0] C_150_ candidate structures, higher sample quantities (1–5 mg) for the early eluting species (Isomer I) would be needed for X-ray crystallographic structure elucidation. It would be challenging for HR-TEM to distinguish between the [9,0] C_150_-D_3h_ and [9,0] C_150_-D_3_ structures. In stark contrast, note that for the structural assignment of [5,5] C_130_-D_5h_, HR-TEM agreed with the anisotropic polarizability method for structural assignment.^10^ In the C_130_ TEM study, the straight row of tubular hexagons was observed at high resolution.

Similarly, efforts to obtain a highly purified sample of [5,5] C_150_ for atomic resolution HR-TEM are currently underway to verify isomer II as [5,5] C_150_-D_5h_. Although resolution of end-cap pentagons and hexagons would be challenging, the [5,5] C_150_ isomer would unambiguously show its signature straight chain of hexagons down its tubular belt.

The Raman G band is significantly broadened for [5,5] C_150_-D_5h_ and is shifted to lower wavenumbers (Figure 7b).^21–24^ Because of this excessive broadening and shift to lower wavenumbers, the G band maxima for [5,5] C_150_-D_5h_ are at 1551 and 1588 cm^–1^ versus 1596 and 1610 cm^–1^, for [5,5] C_120_-D_5d_ (Figure 7a). Thus, the [5,5] C_150_-D_5h_ Raman G band is more significantly broadened and the G^–^ band is ∼40 cm^–1^ at lower wavenumbers as predicted by Dresselhaus et al.^25^

**Figure 7:**
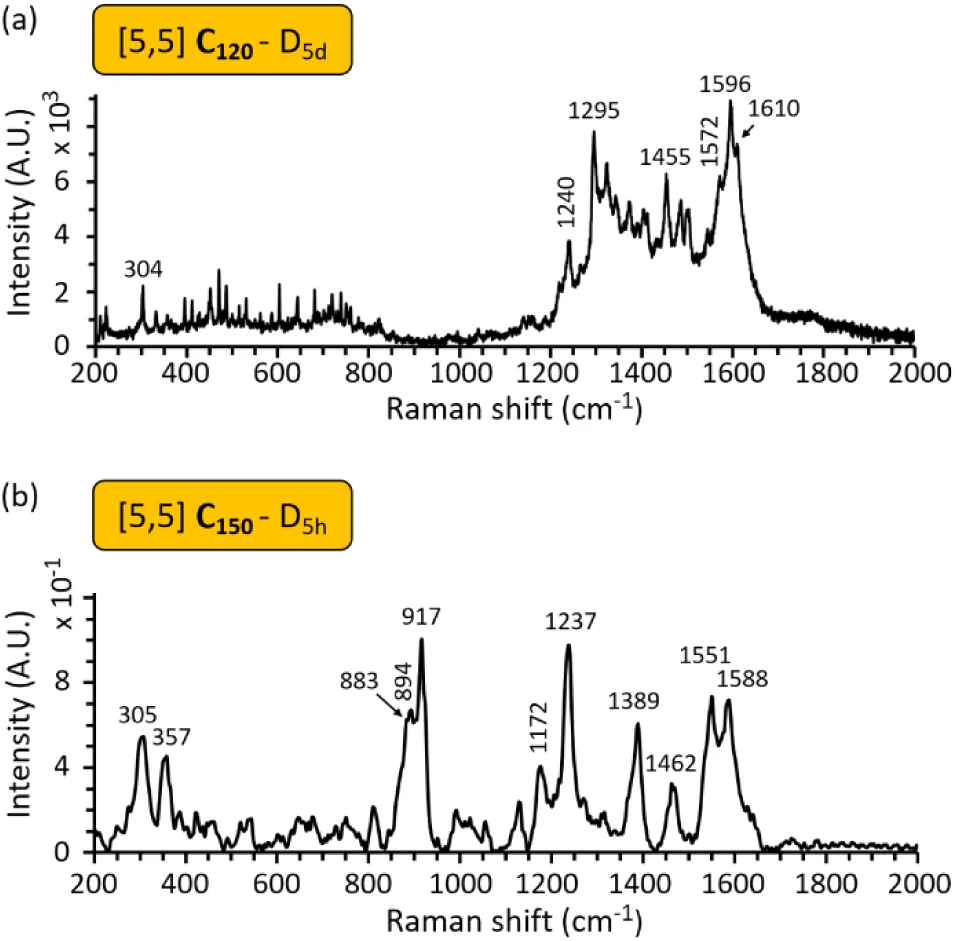
Raman spectra comparison of (a) [5,5] C_120_-D_5d_ versus (b) [5,5] C_150_-D_5h_.

In addition, there is a broad RBM (Radial Breathing Mode) peak centered at 357 cm^–1^ for [5,5] C_150_-D_5h_ which ranges from ∼320–375 cm^–1^ as illustrated in Figure 7b. It should be noted that [5,5] C_150_-D_5h_ possesses 40 end-cap carbons that are part of the 130 *π* tubular belt carbons (Figures 1, 6). The value of 130 was obtained by including 20 carbon atoms from each hemispherical C_30_ end-cap (i.e., 90 fullertube belt +20 left cap +20 right cap).

This range of ∼320–375 cm^–1^ is in excellent agreement with the observed experimental RBM peak at 357 cm^–1^. Assuming the 130 *π* tubular belt carbons in [5,5] C_150_-D_5h_ is sufficiently long enough to use eq 1 by Dresselhaus et al.,^25^ then this would lead to a calculated value of 345 cm^–1^ for experimental comparison (357 cm^–1^). Araujo et al.^26^ have also published similar work relating RBM frequency and tubular diameter for single-wall carbon nanotubes. The presence of a dog-bone structural shape is consistent with the Raman shift to 357 cm^–1^ in Figure 7b. See Supporting Information for the calculation details.

1 $$\omega_{\mathrm{RBM}}=\frac{227}{d_{t}}\sqrt{1+C_{e}{d_{t}}^{2}}$$

The [5,5] C_150_-D_5h_ is the first isolated fullertube to exhibit this dog-bone shape when compared with [5,5] C_120_-D_5d_ (Figures 1 and 6a). The [5,5] C_150_-D_5h_ exhibits an average diameter of 0.668 nm for all 90 [5,5] carbons, Figure 6b.

Guldi and co-workers^27^ predicted that based on HOMO–LUMO gaps, the D_3h_ fullertube isomers should be more stable than the D_3_ isomer. This result is also consistent with our DFT calculations of a smaller HOMO–LUMO gap of 1.025 eV for [9,0] C_150_-D_3_ compared to the higher band gap of 1.525 eV for [9,0] C_150_-D_3h_. See SI and Figure 6. In short, we believe definitive assignment of the two [9,0] candidate C_150_ structures for the 12.3 min. (Isomer I) requires further characterization and future work.

## Conclusion

From a pool of 335,568 IPR isomers, the experimental and *in silico* data support a structure for isomer II as a [5,5] C_150_-D_5h_ molecule isolated in high purity. This D_5h_ assignment of a C_150_ fullertube structure is a strong prediction pending future direct imaging confirmation with atomic resolution TEM. In conjunction with prior fullertube studies, there is experimental evidence that a [5,5] family of 5-fold fullertubes emerges from the aminopropanol chemical reactivity protocol based on both experimental and *in silico* characterization of [5,5] C_90_-D_5h_,^16,17,19^ [5,5] C_100_-D_5d_,^17^ [5,5] C_120_-D_5d_,^11^ [5,5] C_130_-D_5h_^10^ and now [5,5] C_150_-D_5h_. A second C_150_ isomer was also isolated in this study, and this isomer I is *tentatively* structurally identified as either [9,0] C_150_-D_3h_ or [9,0] C_150_-D_3_ species – pending future X-ray crystallography or high-resolution single molecule TEM. When milligram quantities are available beyond the current microgram amounts, X-ray crystallography can validate the predicted structural assignments herein. Nevertheless, this ACS Omega work serves as a harbinger of traditional methods of structural elucidation, but with an established polarizability and DFT paradigm requiring submilligram samples for predicting structural assignments. Experimental mass spectral evidence suggests that fullertube families may extend to longer C_180_ and C_200_ fullertubes, yet to be isolated. Although HOMO–LUMO gaps tend to decrease with increasing carbon number for this [5,5] family, nevertheless, the fullertubes isolated to date have reasonable HOMO–LUMO gaps, with the smallest gap corresponding to only ∼1 eV for [5,5] C_150_-D_5h_. The [5,5] C_90_, C_100_, C_120_, C_130_, and C_150_ fullertubes reported herein serve as a new and separate class of “bridging” all-carbon molecules between fullerenes and single-walled nanotubes (SWCNTs). These new *soluble* C_150_ *molecules* represent a pristine carbon allotrope which can be prepared in higher purity (greater than 99%) for benchmark studies and allows a unique opportunity for direct spectral comparisons with [5,5] SWCNTs.^28^

## Supplementary Material



## Notes

Experimental section; DFT (density functional theory) calculations; computational details; XPS, x, y, and z coordinates; isomer distributions for C_120_, C_150_, C_180_, and C_200_; and candidate structures (PDF)

## ∥

University of Texas, Department of Chemistry, 105 E 24th St, Austin, TX, 78712, USA

## ⊥

University of California, Department of Chemistry, 1102 Natural Sciences II, Irvine, CA, 92697, USA

## #

University of Delaware, Surface Analysis Facility, 023/026 Lammot DuPont Laboratory, Newark, Delaware 19716, USA

## Notes

In Memoriam: Harry Conrad Dorn (August 13, 1943–August 6, 2026) Since joining Virginia Tech in 1974, Harry taught chemistry and mentored generations of future scientists. His initial research at Virginia Tech focused on developing online HPLC-NMR detection and dynamic nuclear polarization. From 1990 to 2026, Harry made seminal contributions to fullerenes, metallofullerenes, and fullertubes, publishing high-impact papers in Nature, Science, Nature Chemistry, and JACS. Throughout his fruitful career, Harry was deeply appreciative of his former students, collaborators, and family for their unwavering support. This ACS Omega manuscript represents one of Harry’s last two scientific contributions. At the time of his death, Harry had 2 papers under editorial review: this one in ACS Omega and JACS Au (accepted and published online). For both papers, Harry was a corresponding author who actively participated in research discussions, wrote text, and was involved with the entire submission process, reviewer responses, and requested revisions until his final days. Just as “Omega” is the final letter in the Greek alphabet, this ACS Omega paper symbolizes the final words that Harry wrote in his 83 years of life. Harry’s life represented a Himalayan climb to the mountaintop; ironically, the cover art is coincidentally Himalayan-themed with his latest discovery of C_150_ new carbon molecule trekking at the peak.

## Abbreviations

IPR: isolated pentagon rule
LDI-TOF: laser desorption ionization–time-of-flight
HPLC: high-performance/high-pressure liquid chromatography
DFT: density functional theory
HR-TEM: high resolution–transmission electron microscopy
STM: scanning tunneling microscopy
RBM: radial breathing mode
fwhm: full width half height
