# Supplementary material for: Experimental Evidence for the Missing and Theoretically Predicted [5,5] C150-D5h Fullertube and Isolation of a Second C150 Isomer
Source: ACS Omega. 2026 Oct 25;11(43):66916–22. doi: 10.1021/acsomega.6c06857 (PMC13737977; doi:10.1021/acsomega.6c06857)
Supplement: Supplementary file 1 [file ao6c06857_si_001.pdf]

Supporting Information

Experimental Evidence for the Missing and Theoretically Predicted [5,5] C_{150}-D_{5h} Fullertube and Isolation of a Second C_{150} Isomer

Xiaoyang Liu[1], Kevin Cover[1], Rong Huang[1], Ryan M. Koenig[2], Hannah M. Franklin,[2] Xu Feng[1], Steven Stevenson*[2,3] and Harry C. Dorn*[1]

1) Department of Chemistry, Virginia Polytechnic and State University, Blacksburg, Virginia 24060, USA

2) Department of Chemistry and Biochemistry, Purdue University Fort Wayne, Fort Wayne, Indiana 46805, USA

3) FIRST Molecules Research Center, Purdue University Fort Wayne, Fort Wayne, Indiana 46805, USA

SI1. Experimental

SI1a: Chemical Separation of Fullertubes with NH_2PrOH

Stage 1: 2500 mg of soluble fullerene extract (*e.g.,* electric-arc or flame method) was dissolved in 1200 mL of toluene in a 2 L beaker (Fig. SI1a). After dissolving, 45 mL of freshly opened 3-amino-1-propanol was added to this vigorously stirring solution. After reaction for 1 h, the stirring was stopped, and the mixture settled into two layers (~30 minutes). Unreacted fullertubes remain in the upper layer (organic phase) of the reaction beaker. This upper layer was poured into a 2-L separatory funnel containing 300 mL of deionized water. This organic phase was washed five times with distilled water, next with 200 mL of 0.1 M HCl, and then five additional times with distilled water. The organic layer was transferred to a round bottom flask for solvent removal in a rotary evaporator. Using this procedure, this sample residue (*e.g.*, 51 mg) is enriched in fullertubes. See Fig. SI1b.

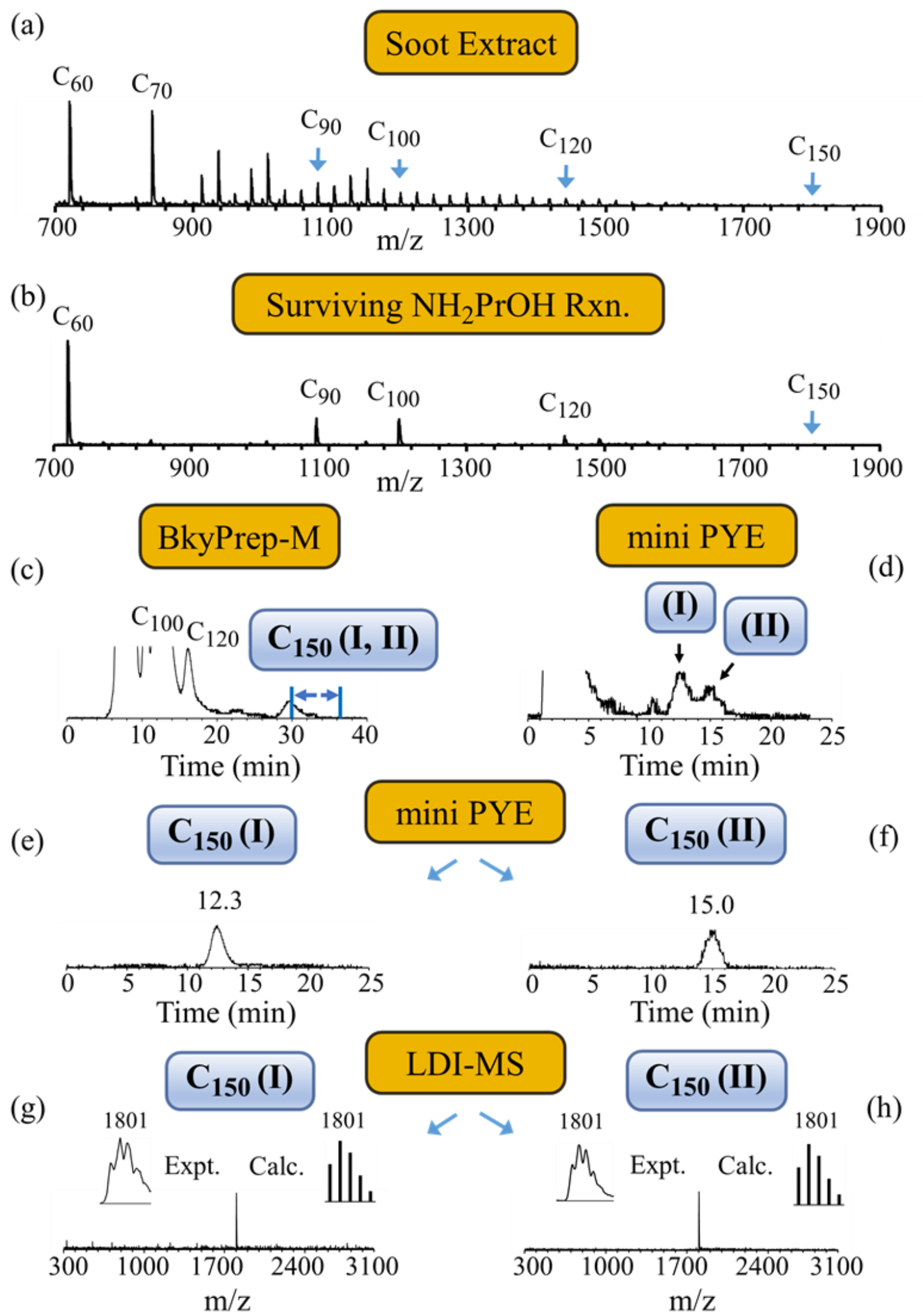

Figure SI1. LDI mass spectra of (a) carbon soot extract, (b) species remaining in solution after addition of NH_2PrOH. (c) HPLC chromatogram corresponding to (b) on a BuckyPrep-M, 1.0 mL/min *o*-xylene. (d) Chromatogram obtained after injection of the fraction collected from the region shown in (c) but now injected into a miniPYE column (2.0 mm x 150 mm). Final HPLC chromatograms and LDI mass spectra are shown for purified isomer I, C_{150}-D_3(2) and/or C_{150}-D_{3h}(3), (e.g) and for (f,h) isomer II, [5,5] C_{150}-D_{5h}(1), 0.5 mL/min o-xylene. The final HPLC chromatogram (f) and corresponding LDI mass spectrum (g) support the isolation of [5,5] C_{150}-D_{5h}(1).

SI1b: HPLC Purification of C_{150} Isomers (*e.g.*, [5,5] versus [9,0])

Stage 2:

<u>BuckyPrep-M Separations</u>

This 51 mg sample containing fullertubes was dissolved in *o*-xylene, PTFE filtered, and subsequently injected into a 4.6 mm x 250 mm BuckyPrep-M column. The objective was obtaining a C_{150} eluting fraction (Fig SI1c). HPLC conditions were 1.0 ml/min *o*-xylene with UV-vis PDA detection at 596 nm.

<u>PYE Separations</u>

Isolation of [5,5] C_{150}-D_{5h}(1) and [9,0] C_{150} Isomers

For this C_{150} isolation work, we used a shorter length and thinner HPLC column (2.0 mm x 100 mm). Specifically, the 30-36 min C_{150} fraction shown in Fig S1c was subsequently injected into this mini-PYE column for HPLC distinguishing of the earlier eluting C_{150} isomer I (12.3 min) and later eluting C_{150} isomer II (15.0 min) as shown in Fig. SI1d. The final HPLC chromatograms for purified isomer I, C_{150}-D_3(2) and/or C_{150}-D_{3h}(3) are shown in Fig. SI1(e,g) versus [5,5] C_{150}-D_{5h}(1) fullertube (isomer II) shown in Fig. SI1(f,h). Quantities of C_{150} were too small to allow adequate measurement of absorption or emission spectra.

SI2: Description of Computational Program and Characterization Procedures: Density Functional theory (DFT) based computations were performed using a Gaussian 09 package. All molecules described below were fully optimized at B3LYP/6-31G(d) level. The electron densities were calculated at the same level and plotted by GaussView. The polarizability was calculated using Gaussian 09 package at B3LYP/6-31G(d) level and the bond polarizabilities are computed based on the C atom coordinates and the α_{xx}, α_{yy} and α_{zz}(**SI3**). The XPS measurements were performed on a PHI VersaProbe III scanning XPS microscope equipped with a monochromatic Al K-alpha X-ray source (1486.6 eV). A dedicated hot sample plate operating up to 800 °C was used to perform sample measurements at 300 °C. High-resolution XPS Spectra were acquired over a 100 µm × 100 µm sample area using 26 eV pass energy, which gives a Ag $3d_{5/2}$ full width at half maximum of 0.57 eV. All binding energies were referenced to the Au $4f_{7/2}$ peak at 84.0 eV.

SI3. C_{150} High Symmetry IPR Candidates and DFT Calculated Properties

**C_{150}-D_5(335566)**		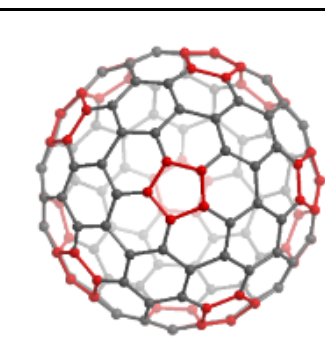
Avg. Polarizability (Å^3)	220.144	
Anisotropic Polarizability (Å^3)	45.503	
HOMO-LUMO gap (eV)	1.619	

**C_{150}-D_{5}(335569)**	
Avg. Polarizability (Å^3)	243.92
Anisotropic Polarizability (Å^3)	31.338
HOMO-LUMO gap (eV)	0.562

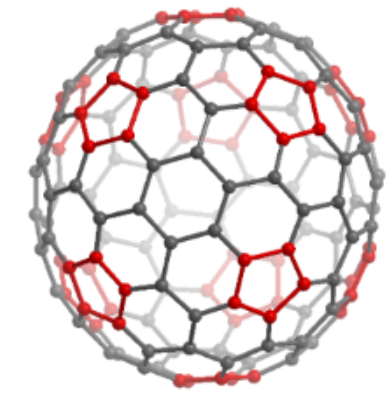

**C_{150}-D_{5h}(335568)**	
Avg. Polarizability (Å^3)	227.958
Anisotropic Polarizability (Å^3)	78.212
HOMO-LUMO gap (eV)	0.838

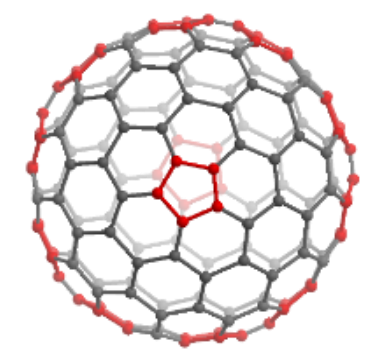

**C_{150}-D_{5h}(333052)**	
Avg. Polarizability (Å^3)	214.877
Anisotropic Polarizability (Å^3)	37.402
HOMO-LUMO gap (eV)	2.213

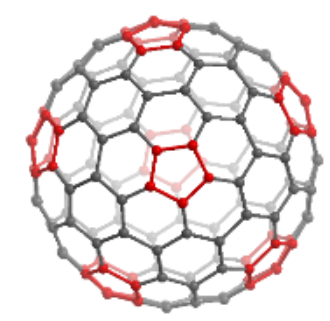

**C_{150}-D_{5h}(1)**	[5,5]
Avg. Polarizability (Å^3)	244.592
Anisotropic Polarizability (Å^3)	255.507
HOMO-LUMO gap (eV)	1.150

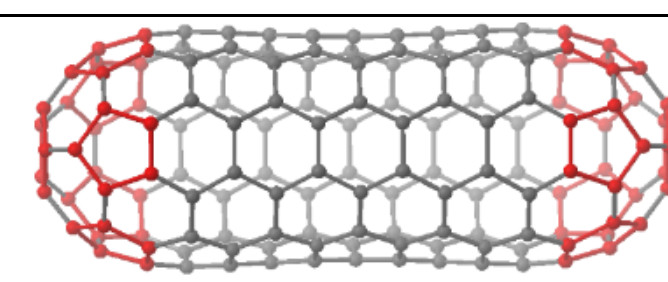

**C_{150}-D_{3}(2)**	[9,0]
Avg. Polarizability (Å^3)	243.144
Anisotropic Polarizability (Å^3)	238.827
HOMO-LUMO gap (eV)	1.025

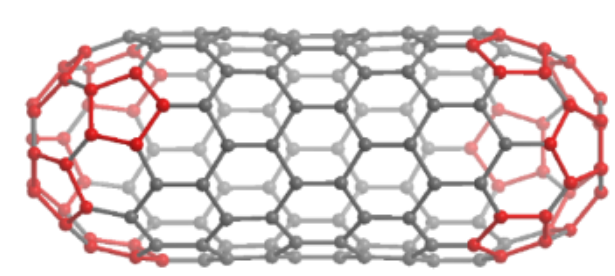

**C_{150}-D_{3}(80339)**	
Avg. Polarizability (Å^3)	229.227
Anisotropic Polarizability (Å^3)	90.743
HOMO-LUMO gap (eV)	0.788

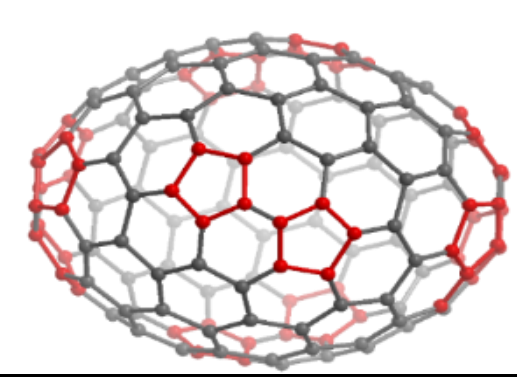

**C_{150}-D_{3}(335545)**	
Avg. Polarizability (Å^3)	227.153
Anisotropic Polarizability (Å^3)	29.468
HOMO-LUMO gap (eV)	1.159

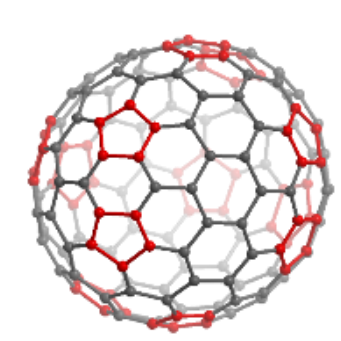

**C_{150}-D_3(325654)**		
Avg. Polarizability (Å^3)	220.755	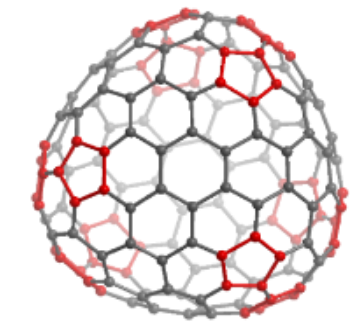
Anisotropic Polarizability (Å^3)	50.894	
HOMO-LUMO gap (eV)	1.408	

**C_{150}-D_3(77150)**		
Avg. Polarizability (Å^3)	216.898	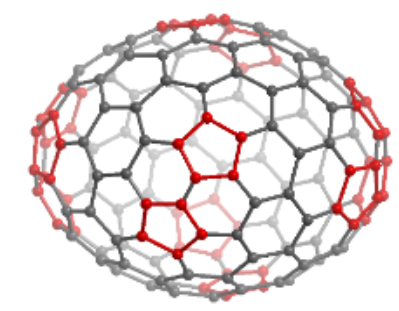
Anisotropic Polarizability (Å^3)	56.837	
HOMO-LUMO gap (eV)	2.088	

**C_{150}-D_3(17039)**		
Avg. Polarizability (Å^3)	254.412	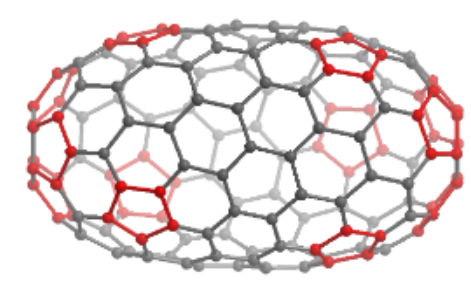
Anisotropic Polarizability (Å^3)	157.035	
HOMO-LUMO gap (eV)	0.519	

**C_{150}-D_3(323057)**		
Avg. Polarizability (Å^3)	223.054	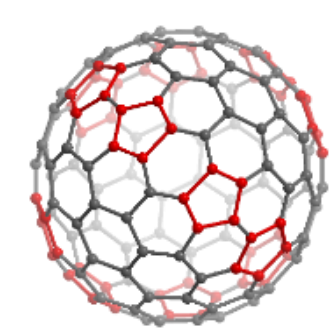
Anisotropic Polarizability (Å^3)	3.086	
HOMO-LUMO gap (eV)	1.432	

**C_{150}-D_3(172152)**		
Avg. Polarizability (Å^3)	221.586	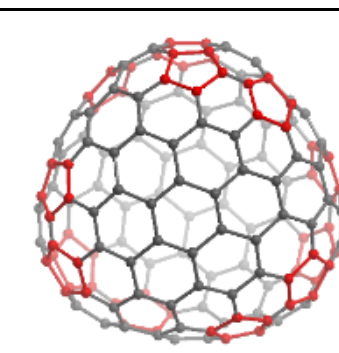
Anisotropic Polarizability (Å^3)	93.007	
HOMO-LUMO gap (eV)	1.590	

**C_{150}-D_3(333053)**		
Avg. Polarizability (Å^3)	215.098	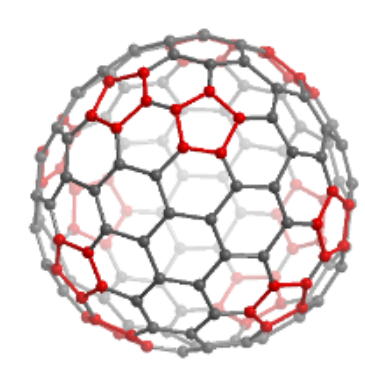
Anisotropic Polarizability (Å^3)	15.617	
HOMO-LUMO gap (eV)	2.222	

**C_{150}-D_3(334416)**		
Avg. Polarizability (Å^3)	261.758	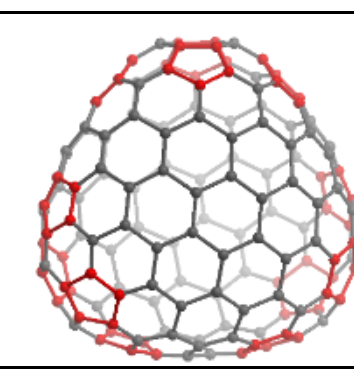
Anisotropic Polarizability (Å^3)	155.212	
HOMO-LUMO gap (eV)	0.488	

**C_{150}-D_{3h}(3)**	[9,0]	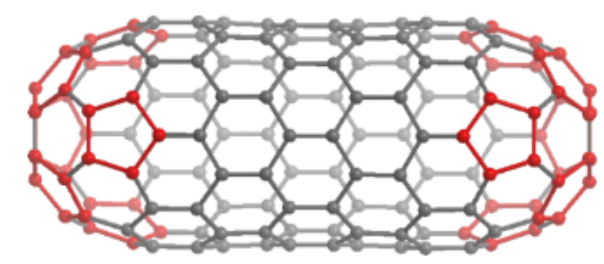
Avg. Polarizability (Å^3)	241.425	
Anisotropic Polarizability (Å^3)	238.782	
HOMO-LUMO gap (eV)	1.525	

**C_{150}-D_{3h}(163532)**		. 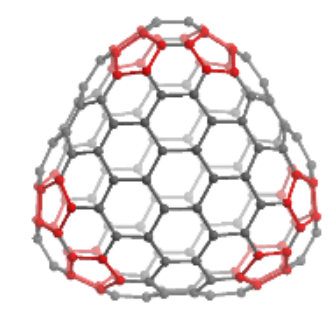
Avg. Polarizability (Å^3)	222.637	
Anisotropic Polarizability (Å^3)	88.292	
HOMO-LUMO gap (eV)	1.858	

SI4: IPR Allowed C_{120}, C_{150}, C_{180} and C_{200} Isomer Distributions[1]

a) IPR C_{120} Isomer Distribution

C_{120}	
symmetry	Isomer count
C_1	9,904
C_2	612
C_{2h}	5
C_{2v}	25
C_3	1
C_{3v}	1
C_i	1
C_s	180
D_2	29
D_{2d}	1
D_{2h}	3
D_3	4
D_5	1
D_{5d}	3
D_{5h}	1
D_6	1
D_{6d}	1
T_d	1

b) IPR C_{150} Isomer Distribution

C_{150}	
symmetry	Isomer count
C_1	331,710
C_2	2792
C_{2v}	57
C_3	7
C_{3v}	4
C_s	982
D_3	10
D_{3h}	2
D_5	2
D_{5h}	3

c) C_{180} IPR Isomer Distribution

C_{180}	
Symmetry	Isomer count
C_1	4,051,036
C_2	17,134
C_{2h}	37
C_{2v}	96
C_3	12
C_{3v}	4
C_i	50
C_s	3,115
D_2	286
D_{2d}	12
D_{2h}	5
D_3	15
D_{3d}	2
D_{3h}	4
D_5	5
D_{5d}	1
D_6	2
D_{6h}	3
I_h	1
S_4	12
sum	4,071,832

d) C_{200} IPR Isomer Distribution

C_{200}	
Symmetry	Isomer count
C_1	15,612,518
C_2	36,317
C_{2h}	58
C_{2v}	163
C_3	126
C_{3v}	2
C_i	97
C_s	5,982
D_2	330
D_{2d}	3
D_{2h}	4
D_3	51
D_{3d}	2
D_{3h}	1
D_5	3
D_{5d}	3
D_{5h}	2
S_4	8
T	1
T_h	1
sum	15,655,672

[1]Fowler, P. W.; Manolopoulos, D. E. *An Atlas of Fullerenes; International series of monographs on chemistry*; 1995.

SI5: XPS Valence Band Region for [5,5] D_{5h}-C_{150} (1) or Isomer II

The final purified [5,5] C_{150} solid samples were washed multiple times with diethyl ether to remove any trace organic non-fullertube contaminants. As a further step to remove any leftover non-fullertube organic traces, *e.g.*, XPS, the deposited [5,5] C_{150} samples were initially heated in the instrument under high temperature to remove any residual organics prior to obtaining final measurements. Confirmation of organic trace removal is demonstrated by examination of manuscript Fig. 3, which shows only fullertube carbon atoms. Meanwhile, the XPS valence band region for purified isomer II, supported as [5,5] D_{5h}-C_{150} (1), is shown below in Figure SI2.

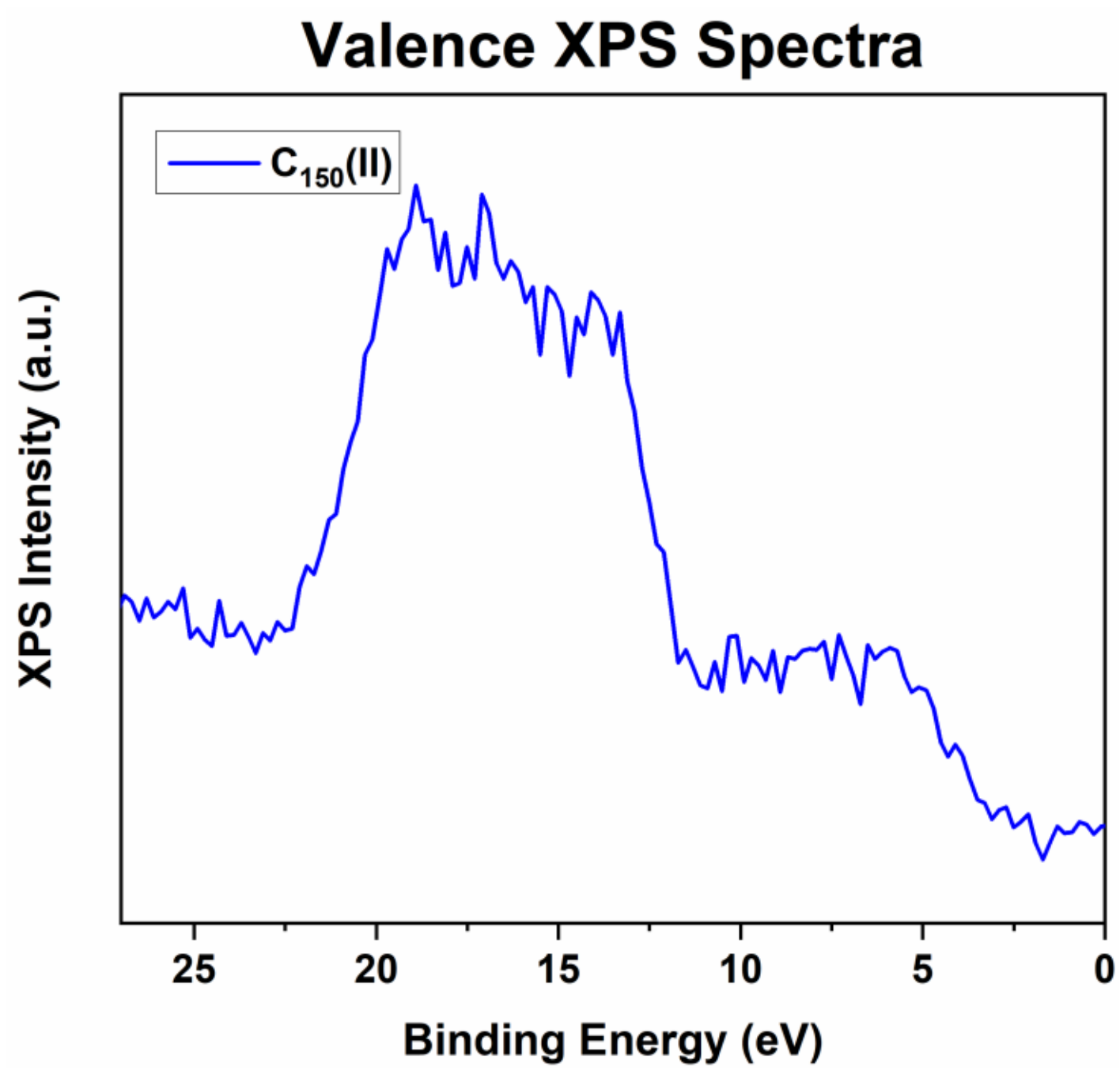

Fig. SI2. Valence Band Region for Isomer II, [5,5] C_{150}-D_{5h}(1)

SI6: Calculated value of ω_{RBM} for [5,5] D_{5h}-C_{150}

From the SI reference below by Dresselhaus et. al.[1], a value for the parameter C_e describing environmental effects of a free-standing nanotube was determined to be 0.07. Using C_e= 0.07 and a tubular diameter (d_t) of 0.668 nm of Fig. 6 in the manuscript, a predicted value of ω_{RBM}. = 345 cm^{-1} can be obtained as a calculated value for a predicted RBM peak appearance.

$$\omega_{RBM} = \frac{227}{d_t} \sqrt{1 + C_e d_t^2}$$

$$\omega_{RBM} = \frac{227}{0.668} \sqrt{1 + 0.07(0.668)^2}$$

$$\omega_{RBM} = 345\ cm^{-1}$$

A similar equation relating ω_{RBM} versus tubular diameter (d_t) was utilized from the Araujo et al.[2] reference below. Using that calculation, a value of ω_{RBM} corresponding to 345 cm^{-1} was obtained.

$$\omega_{RBM} = \frac{227}{d_t}$$

$$\omega_{RBM} = \frac{227}{0.668}$$

$$\omega_{RBM} = 340\ cm^{-1}$$

1. Dresselhaus, M. S.; Jorio, A.; Hofmann, M.; Dresselhaus, G.; Saito, R. Perspectives on Carbon Nanotubes and Graphene Raman Spectroscopy. Nano Lett. 2010, 10, 751.

2. Araujo, P. T.; Maciel, I. O.; Pesce, P. B. C; Pimenta, M. A.; Doorn, S. K.; Qian, H.; Hartschuh, A.; Steiner, M.; Grigorian, L.; Hata, K.; Jorio, A. Nature of the constant factor in the relation between radial breathing mode frequency and tube diameter for single-wall carbon nanotubes, Phys. Rev. B. 2008, 77, 241403.

SI7: x,y, and z coordinates for [5,5] D_{5h}-C_{150}

a) [5,5] D_{5h}-C_{150} (1)

C1	0.00000000	-1.23644000	8.89595900
C2	1.17592400	-0.38208100	8.89595900
C3	0.72676100	1.00030100	8.89595900
C4	1.42104600	1.95590200	8.15004700
C5	0.69536400	2.93901200	7.37110800
C6	1.40095200	3.15616700	6.13369400
C7	0.72798000	3.42949900	4.93166200
C8	1.41413300	3.16314300	3.69475300
C9	0.72104000	3.37803400	2.46044300
C10	1.39800700	3.14694300	1.23650000
C11	0.71103800	3.35615500	0.00000000
C12	1.39800700	3.14694300	-1.23650000
C13	0.72104000	3.37803400	-2.46044300
C14	1.41413300	3.16314300	-3.69475300
C15	0.72798000	3.42949900	-4.93166200
C16	1.40095200	3.15616700	-6.13369400
C17	0.69536400	2.93901200	-7.37110800
C18	1.42104600	1.95590200	-8.15004700
C19	0.72676100	1.00030100	-8.89595900
C20	1.17592400	-0.38208100	-8.89595900
C21	0.00000000	-1.23644000	-8.89595900
C22	0.00000000	-2.41762800	-8.15004700
C23	-1.16494600	-2.78643500	-7.37110800
C24	-0.72175400	-3.37685200	-6.13369400
C25	-1.42686100	-3.20241900	-4.93166200
C26	-0.71519100	-3.39024200	-3.69475300
C27	-1.40222500	-3.15670400	-2.46044300
C28	-0.71871500	-3.36765900	-1.23650000
C29	-1.39745600	-3.13312400	0.00000000
C30	-0.71871500	-3.36765900	1.23650000
C31	-1.40222500	-3.15670400	2.46044300
C32	-0.71519100	-3.39024200	3.69475300
C33	-1.42686100	-3.20241900	4.93166200
C34	-0.72175400	-3.37685200	6.13369400
C35	-1.16494600	-2.78643500	7.37110800
C36	0.00000000	-2.41762800	8.15004700
C37	1.16494600	-2.78643500	7.37110800
C38	2.29006900	-1.96898600	7.37110800

C39	2.29930100	-0.74708800	8.15004700
C40	3.01004500	0.24687400	7.37110800
C41	2.58028700	1.56953500	7.37110800
C42	2.56877500	2.30769400	6.13369400
C43	3.03668900	1.75212300	4.93166200
C44	2.57133600	2.32238500	3.69475300
C45	2.98988800	1.72962000	2.46044300
C46	2.56091300	2.30204300	1.23650000
C47	2.97217000	1.71334600	0.00000000
C48	2.56091300	2.30204300	-1.23650000
C49	2.98988800	1.72962000	-2.46044300
C50	2.57133600	2.32238500	-3.69475300
C51	3.03668900	1.75212300	-4.93166200
C52	2.56877500	2.30769400	-6.13369400
C53	2.58028700	1.56953500	-7.37110800
C54	3.01004500	0.24687400	-7.37110800
C55	2.29930100	-0.74708800	-8.15004700
C56	2.29006900	-1.96898600	-7.37110800
C57	1.16494600	-2.78643500	-7.37110800
C58	0.72175400	-3.37685200	-6.13369400
C59	1.42686100	-3.20241900	-4.93166200
C60	0.71519100	-3.39024200	-3.69475300
C61	1.40222500	-3.15670400	-2.46044300
C62	0.71871500	-3.36765900	-1.23650000
C63	1.39745600	-3.13312400	0.00000000
C64	0.71871500	-3.36765900	1.23650000
C65	1.40222500	-3.15670400	2.46044300
C66	0.71519100	-3.39024200	3.69475300
C67	1.42686100	-3.20241900	4.93166200
C68	0.72175400	-3.37685200	6.13369400
C69	2.60475700	-2.34662700	4.93166200
C70	2.98854300	-1.72993300	6.13369400
C71	3.43461100	-0.35707600	6.13369400
C72	3.48660600	0.36742400	4.93166200
C73	3.44531800	-0.36745500	3.69475300
C74	3.43551500	0.35812000	2.46044300
C75	3.42492900	-0.35712500	1.23650000
C76	3.41161600	0.36087100	0.00000000
C77	3.42492900	-0.35712500	-1.23650000
C78	3.43551500	0.35812000	-2.46044300
C79	3.44531800	-0.36745500	-3.69475300
C80	3.48660600	0.36742400	-4.93166200

C81	3.43461100	-0.35707600	-6.13369400
C82	2.98854300	-1.72993300	-6.13369400
C83	2.60475700	-2.34662700	-4.93166200
C84	3.00330600	-1.72783000	-3.69475300
C85	2.56889200	-2.30907000	-2.46044300
C86	2.98073800	-1.72420300	-1.23650000
C87	2.54794000	-2.29724800	0.00000000
C88	2.98073800	-1.72420300	1.23650000
C89	2.56889200	-2.30907000	2.46044300
C90	3.00330600	-1.72783000	3.69475300
C91	-2.29006900	-1.96898600	7.37110800
C92	-2.98854300	-1.72993300	6.13369400
C93	-2.60475700	-2.34662700	4.93166200
C94	-3.00330600	-1.72783000	3.69475300
C95	-2.56889200	-2.30907000	2.46044300
C96	-2.98073800	-1.72420300	1.23650000
C97	-2.54794000	-2.29724800	0.00000000
C98	-2.98073800	-1.72420300	-1.23650000
C99	-2.56889200	-2.30907000	-2.46044300
C100	-3.00330600	-1.72783000	-3.69475300
C101	-2.60475700	-2.34662700	-4.93166200
C102	-2.98854300	-1.72993300	-6.13369400
C103	-2.29006900	-1.96898600	-7.37110800
C104	-2.29930100	-0.74708800	-8.15004700
C105	-1.17592400	-0.38208100	-8.89595900
C106	-0.72676100	1.00030100	-8.89595900
C107	-1.42104600	1.95590200	-8.15004700
C108	-0.69536400	2.93901200	-7.37110800
C109	-1.40095200	3.15616700	-6.13369400
C110	-0.72798000	3.42949900	-4.93166200
C111	-1.41413300	3.16314300	-3.69475300
C112	-0.72104000	3.37803400	-2.46044300
C113	-1.39800700	3.14694300	-1.23650000
C114	-0.71103800	3.35615500	0.00000000
C115	-1.39800700	3.14694300	1.23650000
C116	-0.72104000	3.37803400	2.46044300
C117	-1.41413300	3.16314300	3.69475300
C118	-0.72798000	3.42949900	4.93166200
C119	-1.40095200	3.15616700	6.13369400
C120	-0.69536400	2.93901200	7.37110800
C121	-1.42104600	1.95590200	8.15004700
C122	-0.72676100	1.00030100	8.89595900

C123	-1.17592400	-0.38208100	8.89595900
C124	-2.29930100	-0.74708800	8.15004700
C125	-3.01004500	0.24687400	7.37110800
C126	-2.58028700	1.56953500	7.37110800
C127	-2.56877500	2.30769400	6.13369400
C128	-3.03668900	1.75212300	4.93166200
C129	-2.57133600	2.32238500	3.69475300
C130	-2.98988800	1.72962000	2.46044300
C131	-2.56091300	2.30204300	1.23650000
C132	-2.97217000	1.71334600	0.00000000
C133	-2.56091300	2.30204300	-1.23650000
C134	-2.98988800	1.72962000	-2.46044300
C135	-2.57133600	2.32238500	-3.69475300
C136	-3.03668900	1.75212300	-4.93166200
C137	-2.56877500	2.30769400	-6.13369400
C138	-2.58028700	1.56953500	-7.37110800
C139	-3.01004500	0.24687400	-7.37110800
C140	-3.43461100	-0.35707600	-6.13369400
C141	-3.48660600	0.36742400	-4.93166200
C142	-3.44531800	-0.36745500	-3.69475300
C143	-3.43551500	0.35812000	-2.46044300
C144	-3.42492900	-0.35712500	-1.23650000
C145	-3.41161600	0.36087100	0.00000000
C146	-3.42492900	-0.35712500	1.23650000
C147	-3.43551500	0.35812000	2.46044300
C148	-3.44531800	-0.36745500	3.69475300
C149	-3.48660600	0.36742400	4.93166200
C150	-3.43461100	-0.35707600	6.13369400

b) [9,0] C_{150} -D_3 (2)

C1	-3.06761500	1.73703700	5.02862200
C2	-3.55478000	0.56991000	5.65625300
C3	-3.68111700	-0.64244600	4.88620600
C4	-3.16226100	-1.73038900	5.65146000
C5	-2.30744200	-2.67081100	5.02432800
C6	-1.16226800	-3.16196600	5.77926400
C7	0.02948900	-3.52515100	5.02862200
C8	1.28383400	-3.36348500	5.65625300
C9	2.39693300	-2.86671800	4.88620600
C10	3.07969100	-1.87340400	5.65146000

C11	3.46671100	-0.66289800	5.02432800
C12	3.31947700	0.57442900	5.77926400
C13	3.03812600	1.78811400	5.02862200
C14	2.27094700	2.79357500	5.65625300
C15	1.28418400	3.50916400	4.88620600
C16	0.08256900	3.60379300	5.65146000
C17	-1.15926900	3.33370900	5.02432800
C18	-2.15720800	2.58753700	5.77926400
C19	-3.09911200	1.78489000	3.58091400
C20	-2.29007800	2.72928300	2.86889500
C21	-1.20098000	3.36550600	3.58354000
C22	-0.00005100	3.59229000	2.83720000
C23	1.26363000	3.45217600	3.50662700
C24	2.33075200	2.75570900	2.83469000
C25	3.09531700	1.79146500	3.58091400
C26	3.50866700	0.61862500	2.86889500
C27	3.51510400	-0.64267400	3.58354000
C28	3.11104000	-1.79610100	2.83720000
C29	2.35785700	-2.82042400	3.50662700
C30	1.22113800	-3.39634500	2.83469000
C31	0.00379600	-3.57635500	3.58091400
C32	-1.21858900	-3.34790700	2.86889500
C33	-2.31412400	-2.72283200	3.58354000
C34	-3.11098900	-1.79618900	2.83720000
C35	-3.62148700	-0.63175300	3.50662700
C36	-3.55189000	0.64063600	2.83469000
C37	-3.52560800	0.63580300	1.42181800
C38	-3.08644600	1.78454100	0.71503700
C39	-2.29576100	2.73310700	1.44297300
C40	-1.22177200	3.35047500	0.71022400
C41	-0.00027600	3.56411500	1.41353000
C42	1.22304100	3.35611600	0.70500200
C43	2.31342600	2.73536400	1.42181800
C44	3.08868100	1.78067000	0.71503700
C45	3.51482000	0.62163400	1.44297300
C46	3.51248300	-0.61715200	0.71022400
C47	3.08675200	-1.78181900	1.41353000
C48	2.29496100	-2.73724300	0.70500200
C49	1.21218200	-3.37116800	1.42181800
C50	-0.00223500	-3.56521100	0.71503700
C51	-1.21905900	-3.35474100	1.44297300
C52	-2.29071100	-2.73332300	0.71022400

C53	-3.08647600	-1.78229700	1.41353000
C54	-3.51800300	-0.61887300	0.70500200
C55	-3.51248300	-0.61715200	-0.71022400
C56	-3.51482000	0.62163400	-1.44297300
C57	-3.08868100	1.78067000	-0.71503700
C58	-2.31342600	2.73536400	-1.42181800
C59	-1.22304100	3.35611600	-0.70500200
C60	0.00027600	3.56411500	-1.41353000
C61	1.22177200	3.35047500	-0.71022400
C62	2.29576100	2.73310700	-1.44297300
C63	3.08644600	1.78454100	-0.71503700
C64	3.52560800	0.63580300	-1.42181800
C65	3.51800300	-0.61887300	-0.70500200
C66	3.08647600	-1.78229700	-1.41353000
C67	2.29071100	-2.73332300	-0.71022400
C68	1.21905900	-3.35474100	-1.44297300
C69	0.00223500	-3.56521100	-0.71503700
C70	-1.21218200	-3.37116800	-1.42181800
C71	-2.29496100	-2.73724300	-0.70500200
C72	-3.08675200	-1.78181900	-1.41353000
C73	-3.11104000	-1.79610100	-2.83720000
C74	-3.51510400	-0.64267400	-3.58354000
C75	-3.50866700	0.61862500	-2.86889500
C76	-3.09531700	1.79146500	-3.58091400
C77	-2.33075200	2.75570900	-2.83469000
C78	-1.26363000	3.45217600	-3.50662700
C79	0.00005100	3.59229000	-2.83720000
C80	1.20098000	3.36550600	-3.58354000
C81	2.29007800	2.72928300	-2.86889500
C82	3.09911200	1.78489000	-3.58091400
C83	3.55189000	0.64063600	-2.83469000
C84	3.62148700	-0.63175300	-3.50662700
C85	3.11098900	-1.79618900	-2.83720000
C86	2.31412400	-2.72283200	-3.58354000
C87	1.21858900	-3.34790700	-2.86889500
C88	-0.00379600	-3.57635500	-3.58091400
C89	-1.22113800	-3.39634500	-2.83469000
C90	-2.35785700	-2.82042400	-3.50662700
C91	-2.39693300	-2.86671800	-4.88620600
C92	-3.07969100	-1.87340400	-5.65146000
C93	-3.46671100	-0.66289800	-5.02432800
C94	-3.31947700	0.57442900	-5.77926400

C95	-3.03812600	1.78811400	-5.02862200
C96	-2.27094700	2.79357500	-5.65625300
C97	-1.28418400	3.50916400	-4.88620600
C98	-0.08256900	3.60379300	-5.65146000
C99	1.15926900	3.33370900	-5.02432800
C100	2.15720800	2.58753700	-5.77926400
C101	3.06761500	1.73703700	-5.02862200
C102	3.55478000	0.56991000	-5.65625300
C103	3.68111700	-0.64244600	-4.88620600
C104	3.16226100	-1.73038900	-5.65146000
C105	2.30744200	-2.67081100	-5.02432800
C106	1.16226800	-3.16196600	-5.77926400
C107	-0.02948900	-3.52515100	-5.02862200
C108	-1.28383400	-3.36348500	-5.65625300
C109	-1.41685800	-2.84115700	-6.97915700
C110	-2.53372900	-1.91035100	-6.97934600
C111	-2.47130100	-0.75266000	-7.72897000
C112	-2.89590600	0.49977000	-7.13730600
C113	-2.08462800	1.53131700	-7.73134800
C114	-1.75208500	2.64761400	-6.97915700
C115	-0.38754800	3.14944900	-6.97934600
C116	0.58382800	2.51653900	-7.72897000
C117	1.88076600	2.25804300	-7.13730600
C118	2.36847300	1.03968200	-7.73134800
C119	3.16894300	0.19354300	-6.97915700
C120	2.92127700	-1.23909800	-6.97934600
C121	1.88747300	-1.76387900	-7.72897000
C122	1.01513900	-2.75781300	-7.13730600
C123	-0.28384500	-2.57099900	-7.73134800
C124	-0.25152900	-1.40000400	-8.59422300
C125	-1.32849300	-0.50631500	-8.59428100
C126	-1.08667500	0.91783200	-8.59422300
C127	0.22576400	1.40366600	-8.59428100
C128	1.33820300	0.48217200	-8.59422300
C129	1.10272800	-0.89735100	-8.59428100
C130	-3.16894300	0.19354300	6.97915700
C131	-2.92127700	-1.23909800	6.97934600
C132	-1.88747300	-1.76387900	7.72897000
C133	-1.01513900	-2.75781300	7.13730600
C134	0.28384500	-2.57099900	7.73134800
C135	1.41685800	-2.84115700	6.97915700
C136	2.53372900	-1.91035100	6.97934600

C137	2.47130100	-0.75266000	7.72897000
C138	2.89590600	0.49977000	7.13730600
C139	2.08462800	1.53131700	7.73134800
C140	1.75208500	2.64761400	6.97915700
C141	0.38754800	3.14944900	6.97934600
C142	-0.58382800	2.51653900	7.72897000
C143	-1.88076600	2.25804300	7.13730600
C144	-2.36847300	1.03968200	7.73134800
C145	-1.33820300	0.48217200	8.59422300
C146	-1.10272800	-0.89735100	8.59428100
C147	0.25152900	-1.40000400	8.59422300
C148	1.32849300	-0.50631500	8.59428100
C149	1.08667500	0.91783200	8.59422300
C150	-0.22576400	1.40366600	8.59428100

c) [9,0] C_{150} - D_{3h} (3)

C1	-2.23628100	2.72756900	5.02752800
C2	-2.91815900	1.68480000	5.78036000
C3	-3.48028500	0.57289200	5.02752800
C4	-3.53447700	-0.69297900	5.65318300
C5	-3.23524700	-1.86787100	4.88367200
C6	-2.36737600	-2.71445800	5.65318300
C7	-1.24400400	-3.30046100	5.02752800
C8	0.00000000	-3.36959900	5.78036000
C9	1.24400400	-3.30046100	5.02752800
C10	2.36737600	-2.71445800	5.65318300
C11	3.23524700	-1.86787100	4.88367200
C12	3.53447700	-0.69297900	5.65318300
C13	3.48028500	0.57289200	5.02752800
C14	2.91815900	1.68480000	5.78036000
C15	2.23628100	2.72756900	5.02752800
C16	1.16710100	3.40743700	5.65318300
C17	0.00000000	3.73574200	4.88367200
C18	-1.16710100	3.40743700	5.65318300
C19	-2.28851600	2.74723100	3.58239700
C20	-1.23984700	3.37734400	2.83499600
C21	0.00000000	3.67511900	3.50567700
C22	1.23984700	3.37734400	2.83499600
C23	2.28851600	2.74723100	3.58239700
C24	3.08940800	1.78367000	2.87055800
C25	3.52343000	0.60829700	3.58239700

C26	3.54478900	-0.61493300	2.83499600
C27	3.18274700	-1.83756000	3.50567700
C28	2.30494200	-2.76241100	2.83499600
C29	1.23491400	-3.35552900	3.58239700
C30	0.00000000	-3.56734100	2.87055800
C31	-1.23491400	-3.35552900	3.58239700
C32	-2.30494200	-2.76241100	2.83499600
C33	-3.18274700	-1.83756000	3.50567700
C34	-3.54478900	-0.61493300	2.83499600
C35	-3.52343000	0.60829700	3.58239700
C36	-3.08940800	1.78367000	2.87055800
C37	-3.09682000	1.78795000	1.44666600
C38	-2.29678700	2.72688000	0.71371100
C39	-1.23268000	3.35098000	1.41587800
C40	0.00000000	3.57264200	0.70313400
C41	1.23268000	3.35098000	1.41587800
C42	2.29678700	2.72688000	0.71371100
C43	3.09682000	1.78795000	1.44666600
C44	3.50994000	0.62563600	0.71371100
C45	3.51837400	-0.60795800	1.41587800
C46	3.09399900	-1.78632100	0.70313400
C47	2.28569400	-2.74302200	1.41587800
C48	1.21315300	-3.35251600	0.71371100
C49	0.00000000	-3.57590000	1.44666600
C50	-1.21315300	-3.35251600	0.71371100
C51	-2.28569400	-2.74302200	1.41587800
C52	-3.09399900	-1.78632100	0.70313400
C53	-3.51837400	-0.60795800	1.41587800
C54	-3.50994000	0.62563600	0.71371100
C55	-3.50994000	0.62563600	-0.71371100
C56	-3.09682000	1.78795000	-1.44666600
C57	-2.29678700	2.72688000	-0.71371100
C58	-1.23268000	3.35098000	-1.41587800
C59	0.00000000	3.57264200	-0.70313400
C60	1.23268000	3.35098000	-1.41587800
C61	2.29678700	2.72688000	-0.71371100
C62	3.09682000	1.78795000	-1.44666600
C63	3.50994000	0.62563600	-0.71371100
C64	3.51837400	-0.60795800	-1.41587800
C65	3.09399900	-1.78632100	-0.70313400
C66	2.28569400	-2.74302200	-1.41587800
C67	1.21315300	-3.35251600	-0.71371100

C68	0.00000000	-3.57590000	-1.44666600
C69	-1.21315300	-3.35251600	-0.71371100
C70	-2.28569400	-2.74302200	-1.41587800
C71	-3.09399900	-1.78632100	-0.70313400
C72	-3.51837400	-0.60795800	-1.41587800
C73	-3.54478900	-0.61493300	-2.83499600
C74	-3.52343000	0.60829700	-3.58239700
C75	-3.08940800	1.78367000	-2.87055800
C76	-2.28851600	2.74723100	-3.58239700
C77	-1.23984700	3.37734400	-2.83499600
C78	0.00000000	3.67511900	-3.50567700
C79	1.23984700	3.37734400	-2.83499600
C80	2.28851600	2.74723100	-3.58239700
C81	3.08940800	1.78367000	-2.87055800
C82	3.52343000	0.60829700	-3.58239700
C83	3.54478900	-0.61493300	-2.83499600
C84	3.18274700	-1.83756000	-3.50567700
C85	2.30494200	-2.76241100	-2.83499600
C86	1.23491400	-3.35552900	-3.58239700
C87	0.00000000	-3.56734100	-2.87055800
C88	-1.23491400	-3.35552900	-3.58239700
C89	-2.30494200	-2.76241100	-2.83499600
C90	-3.18274700	-1.83756000	-3.50567700
C91	-3.23524700	-1.86787100	-4.88367200
C92	-3.53447700	-0.69297900	-5.65318300
C93	-3.48028500	0.57289200	-5.02752800
C94	-2.91815900	1.68480000	-5.78036000
C95	-2.23628100	2.72756900	-5.02752800
C96	-1.16710100	3.40743700	-5.65318300
C97	0.00000000	3.73574200	-4.88367200
C98	1.16710100	3.40743700	-5.65318300
C99	2.23628100	2.72756900	-5.02752800
C100	2.91815900	1.68480000	-5.78036000
C101	3.48028500	0.57289200	-5.02752800
C102	3.53447700	-0.69297900	-5.65318300
C103	3.23524700	-1.86787100	-4.88367200
C104	2.36737600	-2.71445800	-5.65318300
C105	1.24400400	-3.30046100	-5.02752800
C106	0.00000000	-3.36959900	-5.78036000
C107	-1.24400400	-3.30046100	-5.02752800
C108	-2.36737600	-2.71445800	-5.65318300
C109	-2.31097600	-2.17329800	-6.97775600

C110	-3.03761900	-0.91471500	-6.97775600
C111	-2.58070300	0.15250400	-7.73092600
C112	-2.54569900	1.46976000	-7.13852300
C113	-1.42242400	2.15870200	-7.73092600
C114	-0.72664300	3.08801200	-6.97775600
C115	0.72664300	3.08801200	-6.97775600
C116	1.42242400	2.15870200	-7.73092600
C117	2.54569900	1.46976000	-7.13852300
C118	2.58070300	0.15250400	-7.73092600
C119	3.03761900	-0.91471500	-6.97775600
C120	2.31097600	-2.17329800	-6.97775600
C121	1.15827900	-2.31120600	-7.73092600
C122	0.00000000	-2.93952000	-7.13852300
C123	-1.15827900	-2.31120600	-7.73092600
C124	-0.72241100	-1.22536800	-8.59421200
C125	-1.42240600	-0.01294200	-8.59421200
C126	-0.69999400	1.23831100	-8.59421200
C127	0.69999400	1.23831100	-8.59421200
C128	1.42240600	-0.01294200	-8.59421200
C129	0.72241100	-1.22536800	-8.59421200
C130	-3.03761900	-0.91471500	6.97775600
C131	-2.31097600	-2.17329800	6.97775600
C132	-1.15827900	-2.31120600	7.73092600
C133	0.00000000	-2.93952000	7.13852300
C134	1.15827900	-2.31120600	7.73092600
C135	2.31097600	-2.17329800	6.97775600
C136	3.03761900	-0.91471500	6.97775600
C137	2.58070300	0.15250400	7.73092600
C138	2.54569900	1.46976000	7.13852300
C139	1.42242400	2.15870200	7.73092600
C140	0.72664300	3.08801200	6.97775600
C141	-0.72664300	3.08801200	6.97775600
C142	-1.42242400	2.15870200	7.73092600
C143	-2.54569900	1.46976000	7.13852300
C144	-2.58070300	0.15250400	7.73092600
C145	-1.42240600	-0.01294200	8.59421200
C146	-0.72241100	-1.22536800	8.59421200
C147	0.72241100	-1.22536800	8.59421200
C148	1.42240600	-0.01294200	8.59421200
C149	0.69999400	1.23831100	8.59421200
C150	-0.69999400	1.23831100	8.59421200

DFT References

Gaussian 09, Revision A.02, M. J. Frisch, G. W. Trucks, H. B. Schlegel, G. E. Scuseria, M. A. Robb, J. R. Cheeseman, G. Scalmani, V. Barone, G. A. Petersson, H. Nakatsuji, X. Li, M. Caricato, A. Marenich, J. Bloino, B. G. Janesko, R. Gomperts, B. Mennucci, H. P. Hratchian, J. V. Ortiz, A. F. Izmaylov, J. L. Sonnenberg, D. Williams-Young, F. Ding, F. Lipparini, F. Egidi, J. Goings, B. Peng, A. Petrone, T. Henderson, D. Ranasinghe, V. G. Zakrzewski, J. Gao, N. Rega, G. Zheng, W. Liang, M. Hada, M. Ehara, K. Toyota, R. Fukuda, J. Hasegawa, M. Ishida, T. Nakajima, Y. Honda, O. Kitao, H. Nakai, T. Vreven, K. Throssell, J. A. Montgomery, Jr., J. E. Peralta, F. Ogliaro, M. Bearpark, J. J. Heyd, E. Brothers, K. N. Kudin, V. N. Staroverov, T. Keith, R. Kobayashi, J. Normand, K. Raghavachari, A. Rendell, J. C. Burant, S. S. Iyengar, J. Tomasi, M. Cossi, J. M. Millam, M. Klene, C. Adamo, R. Cammi, J. W. Ochterski, R. L. Martin, K. Morokuma, O. Farkas, J. B. Foresman, and D. J. Fox, Gaussian, Inc., Wallingford CT, 2016.

B3LYP
(1) Stephens, P. J.; Devlin, F. J.; Chabalowski, C. F.; Frisch, M. J. Ab Initio Calculation of Vibrational Absorption and Circular Dichroism Spectra Using Density Functional Force Fields. J. Phys. Chem. 1994, 98 (45), 11623–11627. https://nam04.safelinks.protection.out-
look.com/?url=https%3A%2F%2Fdoi.org%2F10.1021%2Fj100096a001&data=05%7C02%7Chdorn%4
0vt.edu%7C272d529959cc44b9569808de99a5ab87%7C6095688410ad40fa863d4f32c1e3a37a%7C0%7
C0%7C639117134028624700%7CUnknown%7CTWFpbGZsb3d8eyJFbXB0eU1hcGkiOnRydWUsI-
lYiOiIwLjAuMDAwMCIsIlAiOiJXaW4zMiIsIkFOIjoiTWFpbCIsIldUI-
joyfQ%3D%3D%7C0%7C%7C%7C&sdata=keh9Hq0up-
scdBoj0u3CGWKxJuR%2B6N0ampkQl%2FdWzNGg%3D&reserved=0.

(2) Becke, A. D. Density-functional Thermochemistry. III. The Role of Exact Exchange. J. Chem. Phys. 1993, 98 (7), 5648–5652. https://nam04.safelinks.protection.out-
look.com/?url=https%3A%2F%2Fdoi.org%2F10.1063%2F1.464913&data=05%7C02%7Chdorn%40vt.
edu%7C272d529959cc44b9569808de99a5ab87%7C6095688410ad40fa863d4f32c1e3a37a%7C0%7C0
%7C639117134028644445%7CUnknown%7CTWFpbGZsb3d8eyJFbXB0eU1hcGkiOnRydWUsIlYi-
OiIwLjAuMDAwMCIsIlAiOiJXaW4zMiIsIkFOIjoiTWFpbCIsIldUI-
joyfQ%3D%3D%7C0%7C%7C%7C&sdata=RNLP2t0c2ES%2FeNwIW2IEWIoCJJVdovkRgHRWbo
%2BSsMw%3D&reserved=0.

(3) Lee, C.; Yang, W.; Parr, R. G. Development of the Colle-Salvetti Correlation-Energy Formula into a Functional of the Electron Density. Phys. Rev. B 1988, 37 (2), 785–789. https://nam04.safelinks.protec-
tion.out-
look.com/?url=https%3A%2F%2Fdoi.org%2F10.1103%2FPhysRevB.37.785&data=05%7C02%7Chdor
n%40vt.edu%7C272d529959cc44b9569808de99a5ab87%7C6095688410ad40fa863d4f32c1e3a37a%7C
0%7C0%7C639117134028659792%7CUnknown%7CTWFpbGZsb3d8eyJFbXB0eU1hcGkiOnRyd-
WUsIlYiOiIwLjAuMDAwMCIsIlAiOiJXaW4zMiIsIkFOIjoiTWFpbCIsIldUI-
joyfQ%3D%3D%7C0%7C%7C%7C&sdata=XNoCxUKHYkllPpM9bimKqlF-
HZmh0isxpFXHxe4mh0Xg%3D&reserved=0.

6-31G(d)
(1) Francl, M. M.; Pietro, W. J.; Hehre, W. J.; Binkley, J. S.; Gordon, M. S.; DeFrees, D. J.; Pople, J. A. Self-consistent Molecular Orbital Methods. XXIII. A Polarization-type Basis Set for Second-row

Elements. J. Chem. Phys. 1982, 77 (7), 3654–3665. https://nam04.safelinks.protection.outlook.com/?url=https%3A%2F%2Fdoi.org%2F10.1063%2F1.444267&data=05%7C02%7Chdorn%40vt.edu%7C272d529959cc44b9569808de99a5ab87%7C6095688410ad40fa863d4f32c1e3a37a%7C0%7C0%7C639117134028674209%7CUnknown%7CTWFpbGZsb3d8eyJFbXB0eU1hcGkiOnRydWUsIlYiOiIwLjAuMDAwMCIsIlAiOiJXaW4zMiIsIkFOIjoiTWFpbCIsIldUIjoyfQ%3D%3D%7C0%7C%7C%7C&sdata=rEmqBcLMdpXeOMy1rIzlDKkLpRD%2FWocskD9Hqv5TicU%3D&reserved=0.
